# Expression of programmed death ligand-1 on tumor cells varies pre and post chemotherapy in non-small cell lung cancer

**DOI:** 10.1038/srep20090

**Published:** 2016-01-29

**Authors:** Jin Sheng, Wenfeng Fang, Juan Yu, Nan Chen, Jianhua Zhan, Yuxiang Ma, Yunpeng Yang, Yanhuang, Hongyun Zhao, Li Zhang

**Affiliations:** 1 State Key laboratory of Oncology in South China, Sun Yat-sen University Cancer Center, Guangzhou, P. R. China; 2 Collaborative Innovation Center for Cancer Medicine, Sun Yat-sen University Cancer Center, Guangzhou, Guangdong, China; 3 Department of Medical Oncology, Sun Yat-sen University Cancer Center, Guangzhou, P. R. China; 4 Department of Medical Oncology, the Fifth Affiliated Hospital of Sun Yat-sen University, Zhuhai, Guangdong, China

## Abstract

The effects of treatments to programmed death ligand-1 (PD-L1) expression is unknown. The aim of this study was to investigate the impact of neoadjuvant chemotherapy (NACT) on PD-L1 expression in non-small cell lung cancer (NSCLC) patients. PD-L1 expression was detected by immunohistochemistry (IHC) method in 32 paired tumor specimens pre and post-NACT. The positivity of PD-L1 on tumor cells (TCs) changed from 75% to 37.5% after NACT (*p* = 0.003). Cases with IHC score of 1, 2, 3 all underwent apparent decrease (p = 0.007). However, no significant changes were observed on tumour-infiltrating immune cells (ICs) (p = 0.337). Subgroup and semiquantitative analyses all presented similar results. Moreover, patients with response to NACT presented significantly reduced PD-L1 expression on TCs (p = 0.004). Although it was not confirmed by the Cox proportional hazard regression model, there was an apparent difference in disease-free-survival (DFS) between negative-to-positive switch of PD-L1 status and the contrary group (median DFS: 9.6 versus 25.9, *p* = 0.005). Our data revealed that antecedent chemotherapy for NSCLC may results in inconsistency of PD-L1 expression. PD-L1 expression is suggested to be monitored around treatment and on serial samples, at least, on the latest tumor specimen.

Global cancer statistics indicate that lung cancer remains the leading cause of cancer-related mortality^1^. Approximately 80–85% of lung cancers are non–small cell lung cancer (NSCLC) and the prognosis remains poor, with an overall 5-year survival rate of only 15%^2^,^3^.

Recent insights into the molecular mechanisms governing the host response to cancer cells has led to the identification of important signaling pathways and checkpoint molecules involved in the anticancer immune response^4^,^5^,^6^. Programmed death ligand-1 (PD-L1, also called B7-H1 or CD274), which is expressed on various cancer and immune cells, plays a crucial role in developing cancer immunoresistance by binding programmed death-1 (PD-1) on T-lymphocytes. The activation of PD-1/PD-L1 axis suppresses T-lymphocytes migration, proliferation and secretion of cytotoxic mediators, and restrains tumor-killing effect^6^,^7^,^8^,^9^,^10^. The resultant T cell suppression contribute to cancer cell immune evasion. Therefore, PD-1 or PD-L1 blockade strategies have been developed to stimulate anti-cancer immunity^10^,^11^. Preclinical data and preliminary results of clinical studies have shown encouraging efficacy and safety profiles of blocking PD-1/PD-L1 pathway with anti-PD-1 or anti-PD-L1 antibodies^10^,^12^,^13^,^14^. Moreover, emerging data have suggested that over-expression of PD-L1 on tumor cells by IHC correlates with inferior prognosis across many cancers but better response to anti-PD-1/PD-L1 therapy^10^,^12^,^14^,^15^,^16^,^17^. Theoretically, the expression of PD-L1 on tumor cells was not consistent. It has been identified that both IFN-gamma secretion and constitutive oncogene activation could stimulate PD-L1 expression^18^,^19^,^20^,^21^. The effect of chemotherapeutic agents on PD-L1 expression is controversial^22^,^23^. Paradoxically, beyond cytotoxic properties, numerous anticancer agents possess the capacity to stimulate host immune system, thus facilitate tumor eradication^24^,^25^,^26^.

Based on above data, the prognostic and predictive value of PD-L1 IHC was assumed to temporally dependent on the antecedent treatment and time of biopsy. We therefore conducted this exploratory analyses with paired NSCLC specimen pre and post-NACT to explore the impact of chemotherapy on PD-L1 expression. The association between PD-L1 change patterns and prognosis were also analyzed.

## Results

### Patient characteristics and treatment outcomes

Finally, 32 patients (17 female) were included for analyses. Baseline demographics and PD-L1 expression, as presented by H-score, are summarized in Table 1. The median age of patients at diagnosis was 56 (range, 36–77) years old. More than half (n = 22, 68.8%) patients never smoked. Most cases were (n = 26, 81.3%) adenocarcinoma. There were 21 patients (65.6%) staged as IIIA, while another 11 (34.4%) were diagnosed with IIIB stage (pT4N0 or N1). We routinely recorded the oncogenetic mutation status. However, there were six cased (18.8%) unidentified due to finite specimen. Eleven patients harbored EGFR sensitive mutations, such as L858R at exon 21 (n = 3, 9.4%) and exon 19 deletion (n = 8, 25%). The reminding fifteen patients (46.9%) were wild type of EGFR-activating mutations. No other driver oncogenetic mutations were detected. Fifteen patients (46.9%) received pemetrexed-based regimen, nine cases (28.1%) were treated by paclitaxel-based chemotherapy. Of note, among eight (25%) patients received EGFR-TKIs, four patients were treated with erlotinib plus chemotherapy (gemcitabine 1,000 mg/m^2^, days 1 and 8 plus cisplatin 75 mg/m^2^, days 1, every 3 weeks). The median tumor size at baseline was 35.0 mm (IQR: 26.3–40.0). After the NACT, the median value decreased to 22.0 (IQR: 18.0–30.3). This change was significant according to the result of Wilcoxon test (p < 0.001). Finally, partial response were found in 18 (56.2%) patients, while 50% patients (n = 16) achieved down-staging after NACT.

### The impact of NACT on PD-L1 expression on TCs and ICs

**Figure 1:**
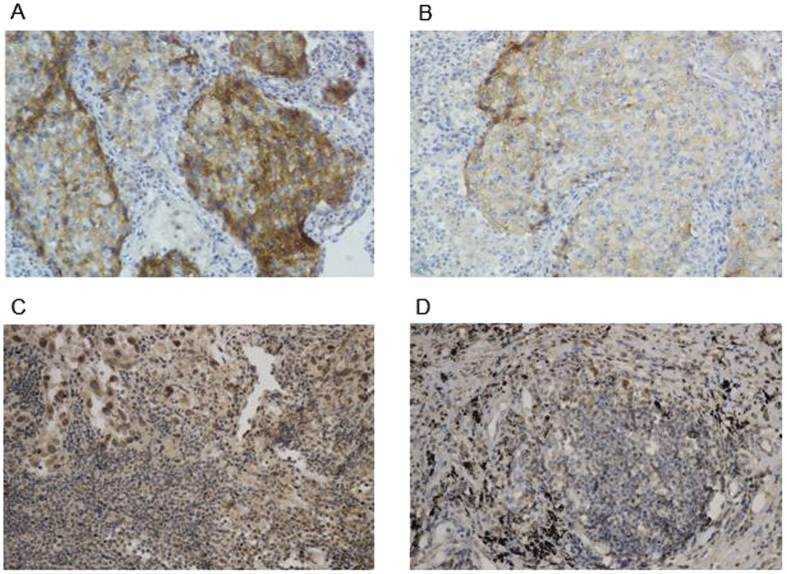
The expression of PD-L1 by immunohistochemical staining with a membranous pattern. Representative staining on TCs at baseline (**A**) and after NACT (**B**). Representative staining on ICs at baseline (**C**) and after NACT (**D**).

Figure 1 illustrated representative PD-1 IHC staining on the membrane of TCs and ICs. We observed no significant differences of PD-L1 expression across various clinicopathological parameters, neither at baseline nor after NACT (Supplementary Table S1 online). The positive rate of PD-L1 on TCs from pre-NACT specimens was 75%, which decreased to 37.5% after NACT (*p* = 0.003). However, a contrary result was found in ICs, whose PD-L1 positive rate increased from 43.8% to 56.2% after NACT, although no statistical significance was reached (*p* = 0.319).

Table 2 summarized the changes of PD-L1 expression on TCs. Subgroup analyses backed up the observed instable PD-L1 positivity satus on TCs around chemoherapy. Of note, the PD-L1 positivity around NACT differed significantly in patients received paclitaxel-based therapy or TKI-based therapy, but not in those received pemetrexed-based regimens.

**Figure 2:**
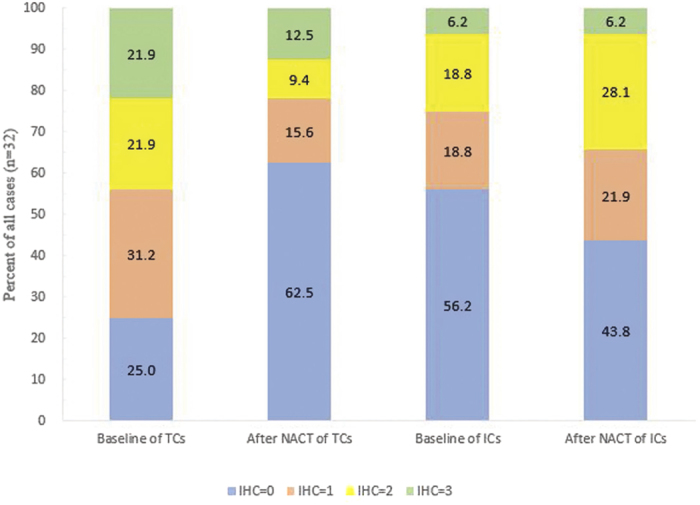
The percent of four PD-L1 status on TCs and ICs pre and post-NACT. Specimens were scored as IHC 0, 1, 2, or 3 if, 1%, ≥1% but <5%, ≥5% but <10%, or ≥10% of cells per area were PD-L1 positive, respectively.

Another perspective is, as shown in Fig. 2, the percents of cases with IHC score of 1, 2, 3 all underwent an apparent decrease after NACT, which also indicates a significant difference of PD-L1 status on TCs around NACT (*p* = 0.007). Subgroup analyses also supported the finding, as summarized in Supplementary Table S2 online. Similarly, this phenomenon did not apply to the result presented on the membrane of ICs (*p* = 0.337).

**Figure 3:**
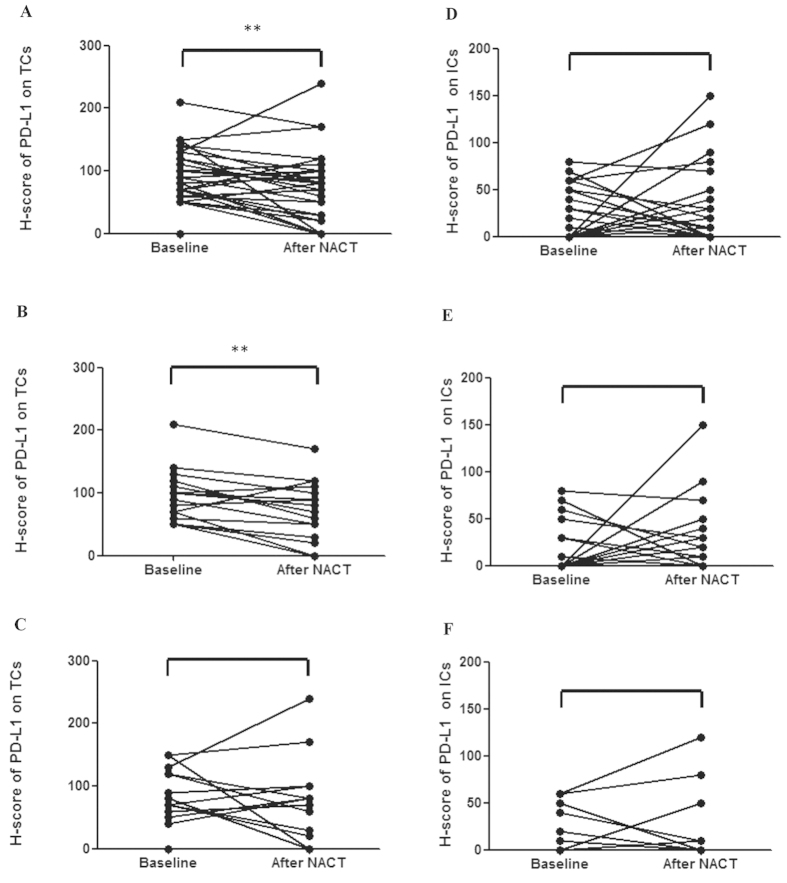
Semiquantative analyses by H-score on TCs and ICs and the correlation with response to NACT. The H-score of PD-L1 on TCs of all patients (**A**), those with response to NACT (**B**) and those failed to acquire objective response (**C**). The H-score of PD-L1 on ICs of all patients (**D**), achieved partial response (**E**) and not (**F**).

Semiquantitative analyses demonstrated that the H-score of PD-L1 on TCs was significantly decreased (median value: 95 versus 75, *p* = 0.005, Fig. 3A) after NACT, which was not observed on ICs (median value: 10 versus 0, *p* = 0.474, Fig. 3D). The results were also summarized in numerical form in Supplementary Table S3 on line. Patients with response to NACT were correlated with significantly reduced PD-L1 expression on TCs (*p* = 0.004, Fig. 3B), but not on ICs (*p* = 0.378, Fig. 3E). For the cases failed to acquire objective response after NACT, no apparent changes of PD-L1 expression were detected on neither TCs nor ICs (*p* = 0.441, 1.000 separately, Fig. 3C and Fig. 3F). In summary, the above semiquantitative data demonstrated that PD-L1 expression is not consistent around NACT, at least on TCs.

### Change patterns of PD-L1 around NACT and the prognosis

**Figure 4:**
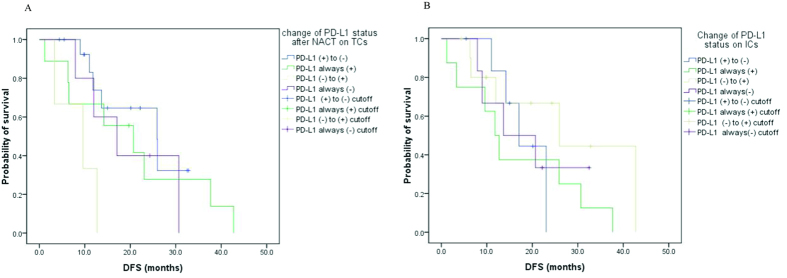
The prognostic value of PD-L1 change patterns for disease-free survival. Four change patterns on TCs (**A**). Corresponding classifications on Ics (**B**).

Finally, we evaluated the prognostic value of PD-L1 change patterns. In general, the median DFS after radical surgery was 20.7 (95% CI: 8.4–33.0) months. As shown in Fig. 4A, the negative-to-positive switch of PD-L1 status was significantly associated with inferior DFS, compared with the contrary group, in which PD-L1 status changed reversely (median DFS: 9.6 versus 25.9, *p* = 0.005). Besides, no significant differences were detected among four change patterns of PD-L1 on ICs (*p* = 0.445, Fig. 4B). To make it more distinct, Kaplan-Meyer curves of PD-L1 negative-to-positive switch and then all other cases were also presented in Supplementary Figure S1 online. Multivariate analyses based on clinical-pathological features and PD-L1 status around NACT failed to support the independent role of PD-L1 status switch. Independent facots associasted with longer DFS included non-smoker and no lymph node invasion (Supplementary Table S4).

## Discussion

The PD-1/PD-L1 axis is one of the crucial mechanisms underlying immune escape of tumor cells. These pathways are currently attractive therapeutic targets for human cancers, including NSCLC. One of the provocative findings accompanied with anti-PD-L1/PD-1 exciting efficacy involves the potential predictive value of PD-L1 expression on tumor cells^20^. So far, there are considerable unsolved issues about the predictive value of PD-L1 expression in NSCLC, considering the technical aspects of tests, dynamic changes, and prognostic implications among other factors^27^,^28^.

Our result demonstrated that the expression of PD-L1 on tumor cells is not consistent for patients with NSCLC. The expression profile was correlated with antecedent chemotherapy. Therefore, the dynamic property of PD-L1 expression demonstrated by our study may provide a possible reason to why negative status from chemo-naive sample finally confer certain responses when PD-1/PD-L1-directed therapy are given in second or later lines^14^.

Many anticancer agents exert immunomodulatory effects on host system in addition to their cytotoxicity^26^. The effects of chemotherapeutic on expression of PD-L1 have been previously explored in breast cancer cells. Zhang *et al.* reported that cytotoxic agents, specifically paclitaxel, etoposide and 5-fluorouracil, could induce PD-L1 surface expression in breast cancer cells, which lead to promoted PD-L1-mediated T cell apoptosis^22^. On the contrary, Ghebeh *et al.* have revealed doxorubicin-dependent down-regulation of cell surface PD-L1^23^. The discrepancy was attribute to heterogeneity among different malignancies and agents. Another reason was cancer cells with high-level PD-L1 expression may present more aggressive potential and vascular invasion^29^, which confers better sensitivity to cytotoxic agents. We observed a different PD-L1 reduction effect among three NACT therapies. It has been reported that PD-L1 expression is increased by EGFR signaling conferred by activating EGFR mutations and the EGFR inhibitor erlotinib could down-regulate PD-L1 expression^19^. Based on our data, patients received TKI or taxane-based therapy tends to reach significant PD-L1 reduction on TCs, whereas pemetrexed-base regimen not. Besides, for patient with response to NACT, we observed a significant decrease of PD-L1 (*p* = 0.004) on TCs, but not in those not responded (*p* = 0.196). To verify the hypothesis, we analyzed the difference of PD-L1 expression on tumor-infiltrating lymphocytes (ICs). No significant alteration was observed, neither in the response group nor non-response group. Of note, whether drug resistance induces PD-L1 expression is largely unknown. In present study, no significant induction of PD-L1 on tumor cells was detected in population exerted no response to NACT. We thereby proposed non-monotonic mechanisms underlying the effect of chemotherapy on PD-L1 expression, including both cytotoxic-related PD-L1 reduction and other signal-associated PD-L1 regulation. It has been identified that constitutive IFN-gamma secretion or oncogene activation will stimulate PD-L1 expression. Besides, PD-L1 up-regulation mediated by IFN-gamma is commonly considered as a classically adaptive way for immune resistance^30^. Surface expression of PD-L1 on tumor cells has been associated with activation of several oncogenic pathways including the p-ERK1/2/p-c-Jun pathway from EGFR activation, the PI3K/Akt and P13K/mTOR pathways as well as the STAT3 pathway from ALK activation in NSCLC^31^,^32^. To sum up, further studies are warranted to explore the intrinsic mechanism of chemotherapy-induced alteration of PD-L1 expression, both on TCs and ICs in NSCLC.

The prognostic value of PD-L1 has also been well-discussed among various cancers. PD-L1 expression on tumor cells correlates with poor clinical prognosis of renal, ovarian cancers, breast cancers^15^,^16^,^17^,^33^. However, the prognostic role of PD-L1 still remains controversial. Konishi *et al.* reviewed that PD-L1 and PD-L2 did not correlate to the prognosis of NSCLC, nor did they relate to other clinically pathological factors^34^. Mu *et al.* reported that high expression of PD-L1 in the primary foci of NSCLC was an independent predictor of poor prognosis^35^. However, Cooper *et al.* revealed that high PD-L1 expression is independently associated with longer overall survival and correlated with high tumor grade and younger patient age^36^. We evaluated whether the change patterns of PD-L1 status correlate with the prognosis after radical resection. No significant differences were detected among four change patterns of PD-L1 on ICs. Moreover, with Kaplan-Meier analyses, the negative-to-positive switch of PD-L1 status was significantly associated with impaired DFS, compared with the contrary group, in which PD-L1 status changed reversely (median DFS: 9.6 versus 25.9, p = 0.005). However, this was not confirmed in Cox proportional hazard regression model. Relatively small samples may contribute to this result. Indeed, comparison of different studies about PD-L1 expression in NSCLC is hindered by discrepant issues, including methodologies, thresholds to determine positivity and clinicopathological differences in cohorts^36^. Our result provided another interpretation that the dynamic feature of PD-L1 and instability affected by anticancer agents may contributes to the prognostic discrepancy among present studies, especially those reporting result from treatment-naive samples and specimens acquired after disease progression.

For patient with pathological N2 NSCLC, the most menacing problem is postoperative recurrence. Thus new therapeutic targets for NSCLC are urgently needed. Alternative strategies such as immunotherapy or combination strategies involved of immunotherapy are now being considered for NSCLC treatment. CheckMate 012 investigate the efficacy and safety of combining anti-PD-1 antibody nivolumab with platinum doublet (n = 56), which achieved ORR as 33–50%, and remarkable 1-year survival rate as 59–87%^37^. Based on these preliminary data, the clinical efficacy of nivolumab in combination with platinum doublets is highly promising. The marginal difference existed among different NACT regimens in this study needed further confirmation and may provide clues for designing a rational combination strategy. A better understanding of immunomodulatory effects of chemotherapy and targeted agents enable the design of more rational combinations with immunotherapy. Whether immunotherapy is beneficial for patient with IIIA /IIIB (pT4N0 or N1) NSCLC as neoadjuvant treatment regimen or adjuvant therapy remains further investigation. In order to incorporate immunotherapy into clinical practice for NSCLC, a better understanding of the PD-L1 biological property is of vital importance.

This study is limited by retrospective feature and relatively small samples. Althouth we present an interesting and relevant finding, further research are warranted to explore the intrinsic mechanism of chemotherapy-induced alteration of PD-L1 expression. Considering the complexity of tumor micro-environment, deep illustration about effects of chemotherapy on tumor infiltrating lymphocytes patterns may provide better interpretation. The alteration of PD-L1 expression confers potentially impact on future treatment decision. Despite certain limitations, our data was the first to demonstrate the inconsistance of PD-L1 around chemotherapy and highlight the need to monitor PD-L1 biological changes around treatment and on serial samples, at least, the latest biopsy.

## Conclusion

Based on our result, the expression of PD-L1 on tumor cells is not consistent around chemotherapy for patients with NSCLC. The value as a predictive biomarker for anti-PD-1 efficacy and future immunotherapeutic decision should be cautious about the inconstancy of PD-L1. PD-L1 expression is suggested to be monitored around treatment and on serial samples, at least, on the latest tumor specimen.

## Patients and Methods

### Patients

All patients were evaluated in a multidisciplinary meeting for treatment strategy setting. Patients pathologically diagnosed with NSCLC and received NACT prior to surgical resection at Sun Yat-sen University Cancer Center (SYSUCC, Guangzhou, China) from January 2010 to March 2014 were screened for eligibility. We only included cases met all of the following conditions: 1). diagnosed as stage IIIA (N2)/IIIB (T4N0-1) by invasive mediastinoscope; 2). received 1-4 cycles neoadjuvant chemotherapy before radical resection; 3). with enough paired tumor tissue samples around NACT for IHC staining of PD-L1.

This retrospective study was approved by the Institutional Review Board of Sun Yat-sen University Cancer Center. All the patients had provided written informed consent before tissue samples were collected. All procedures performed in studies involving human participants were in accordance with the ethical standards of the national research committee and with the 1964 Helsinki Declaration and its later amendments or comparable ethical standards. The studies conducted in laboratory were operated under exploratory research principles.

### Treatment schedule

Patient diagnosed as stage IIIA (N2)/IIIB (T4N0-1) by invasive mediastinoscope were transferred to medical oncology department for neoadjuvant chemotherapy. According to the regimens given to patients, the neoadjuvant chemotherapy (NACT) were classified as three types: 1. Paclitaxel-based NACT, which refers to paclitaxel (175 mg/m^2^ IV, days 1) plus carboplatin (AUC = 6, IV, day 1) or cisplatin (75 mg/m^2^, IV, day 1), every 3 weeks. 2. Pemetrexed-based NACT, including pemetrexed (500 mg/m^2^, IV, day 1) plus cisplatin (75 mg/m^2^, IV, day 1), every 3 weeks. 3. TKI-based NACT, means treatment including erlotinib (150 mg, daily), gefitinib (250 mg, daily) or icotinib (125 mg, three times a day) for a period of at least 4 weeks, stopped 72 hours before surgery. All patients were re-evaluated by radiographic method after two cycles of treatment (four weeks for TKI-based NACT) for resectability. Adjuvant chemotherapy was administrated rouinely after resection, according to the sensitivity to neoadjuvant regimen and pathological type of cancer.

Baseline clinical and pathological features were collected from the electronic medical system. Paired tissue specimens were acquired from baseline biopsy and later surgery. The clinicopathological features included age, gender, smoking status, pathological type, stage (according to the Union for International Cancer Control, the seventh edition), EGFR mutation status and classification of neoadjuvant regimens.

### Immunohistochemistry analysis

The expression of PD-L1 in human NSCLC specimens was performed with IHC staining using rabbit monoclonal anti-human antibody (E1L3N™, Cell Signaling Technology, Danvers, MA, 1:200). Five-μm-thick Sections were cut from the formalin-fixed, paraffin-embedded (FFPE) tumor block and then routinely deparaffined and rehydrated. For antigen retrieval, slides were heated in a microwave oven for 30 minutes in citrate buffer solution (pH = 7.4) and cooled slowly at room temperature for 20 minutes. Then, we blocked the activity of endogenous peroxidase with 3% hydrogen peroxide for 8 minutes. Thereafter, the sections were treated with primary antibodies and incubated for overnight (more than 12 hours). Subsequently, the slides were rinsed in PBS three times and incubated in HRR-linked secondary antibodies. After incubation, slides were washed again with PBS and then visualized using diaminobenzidine. Finally, Mayer’s hematoxylin was used to counterstain the sections and dehydrated and mounted. This mature method was applied in our previous work and detailed described^38^.

Semiquantitative H score (maximum value of 300 corresponding to 100% of tumor cells positive for PD-L1 with an overall staining intensity score of 3) was defined as multiplying the percentage of stained cells by an intensity score (0, absent; 1,weak; 2, moderate; and 3, strong)^19^,^35^. A 5% proportion of membrane-positive TCs which were defined as H-score ≥5 have been used as cutoff for PD-L1 positivity^10^. Specimens were scored as IHC 0, 1, 2, or 3 if, 1%, ≥1% but <5%, ≥5% but <10%, or ≥10% of cells per area were PD-L1 positive, respectively^12^. The expression pattern of PD-L1 on ICs were also detected as aforesaid. Two pathologists were blinded to the clinical or pathological information of these patients and independently assessed the expression of PD-L1. For specimen with heterogeneous result, two pathologists re-evaluated the PD-L1 positivity status to reach a common opinion after consultation. Semiquantitative H score were recorded as the average score.

### Statistical analysis

All the statistical analysis was performed using SPSS 20.0 for Windows (IBM, Armonk, NY). Non-parametric quantitative data were presented as median value and interquartile range (IQR). Wilcoxon or Mann-Whitney method was used for comparision of non-parametric data. Pearson’s chi-squared test or Fisher exact test was used to assess the correlation between changes of PD-L1 expression and clinicopathologic variables. DFS was defined as the time from data of surgery to recurrence. Survival analyses were performed by Kaplan-Meier method and Cox proportional hazard regression model. A two sided *p*-value of <0.05 was considered statistically significant at all situation.

## Additional Information

**How to cite this article**: Sheng, J. *et al.* Expression of programmed death ligand-1 on tumor cells varies pre and post chemotherapy in non-small cell lung cancer. *Sci. Rep.* **6**, 20090; doi: 10.1038/srep20090 (2016).

## Supplementary Material

Supplementary Files

## Figures and Tables

**Table 1:** Baseline demographics and clinical characteristics of patients.

Characteristics	N (Proportion, %)
Total	32
Median Ages (years)	56 (36–77)^a^
Gender	
Female	17 (53.1)
Male	15 (47.9)
Smoking status	
Never smoker	22 (68.8)
Current or ex-smoker	10 (31.2)
Histologic diagnosis	
Adenocarcinoma	26 (81.3)
Squamous carcinoma	5 (15.6)
Others	1(3.1)
Stage	
IIIA (N2)	21 (65.6)
IIIB (T4N0 or 1)	11 (34.4)
Types of EGFR mutation	
L858R	3 (9.4)
19 deletion	8 (25.0)
Wild-type	15 (46.9)
Untested	6 (18.8)
NACT regimen	
Paclitaxel-based	9 (28.1)
Pemetrexed-based	15 (46.9)
TKI-based	8 (25.0)
Down-staging	
Yes	16 (50.0)
No	16 (50.0)
NACT efficacy	
Partial response	18 (56.2)
Stable or progression	14 (43.8)

Note.

^a^Median (range). Abbreviations: *NACT*, neo-adjuvant chemotherapy; *TKI*, tyrosine kinase inhibitors.

**Table 2:** Changes of PD-L1 positivity status on TCs pre and post-neoadjuvant NACT and patient characteristics.

Characteristics	N (%)	Cases with positive PD-L1 on TCs		
		Pre-NACT	Post-NACT	*p*
Age (years)				
<56	16 (50.0)	11 (68.7)	4 (25.00)	0.015
≥56	16 (50.0)	13 (81.3)	8 (50.00)	0.067
Gender				
Female	17 (53.1)	12 (70.6)	6 (35.3)	0.042
Male	15 (47.9)	12 (80.0)	6 (40.0)	0.028
Smoking status				
Never smoker	22 (68.8)	16 (72.7)	9 (40.9)	0.035
Current or ex-smoker	10 (31.2)	8 (80.0)	3 (30.0)	0.029
Histologic diagnosis				
Non-squamous	27 (84.4)	19 (70.4)	11 (40.7)	0.030
Squamous carcinoma	5 (15.6)	5 (100)	1 (20.0)	0.014
Stage				
IIIA (N21)	21 (65.6)	16 (76.2)	6 (28.6)	0.002
IIIB (T4N0 or 1)	11 (34.4)	8 (72.7)	6 (54.5)	0.386
Types of EGFR mutation				
Mutated	11 (34.4)	9 (81.8)	3 (27.3)	0.012
Wild-type	15 (46.9)	11 (73.3)	5 (33.3)	0.031
Untested	6 (18.8)	4 (66.7)	4 (66.7)	1.0
NAC regimen				
Paclitaxel-based	9 (28.1)	8 (88.9)	2 (22.2)	0.006
Pemetrexed-based	15 (46.9)	9 (60.0)	9 (60.0)	1.0
TKI-based	8 (25.0)	7 (87.5)	1 (12.5)	0.004
NACT efficacy				
Partial response	18 (56.2)	17 (94.4)	6 (33.3)	<0.001
Stable or progression	14 (43.8)	7 (50.0)	6 (42.9)	0.712

Abbreviations: *PD-L1*, programmed death-ligand 1; *NACT*, neo-adjuvant chemotherapy; *TCs*, tumor cells; *TKI*, tyrosine kinase inhibitors.

## References

[b1] Parkin D. M., Bray F., Ferlay J. & Pisani P. Global cancer statistics, 2002. CA Cancer J Clin 55 (**2**), 74 (2005). 15761078 10.3322/canjclin.55.2.74

[b2] Jemal A., Siegel R., Xu J. & Ward E. Cancer statistics, 2010. CA Cancer J Clin 60 (**5**), 277 (2010). 20610543 10.3322/caac.20073

[b3] Sun S., Schiller J. H., Spinola M. & Minna J. D. New molecularly targeted therapies for lung cancer. J Clin Invest 117 (**10**), 2740 (2007). 17909619 10.1172/JCI31809 PMC1994616

[b4] McDermott D. F. & Atkins M. B. PD-1 as a potential target in cancer therapy. Cancer Med 2 (**5**), 662 (2013). 24403232 10.1002/cam4.106 PMC3892798

[b5] Keir M. E., Butte M. J., Freeman G. J. & Sharpe A. H. PD-1 and its ligands in tolerance and immunity. Annu Rev Immunol 26, 677 (2008). 18173375 10.1146/annurev.immunol.26.021607.090331 PMC10637733

[b6] Dong H., Zhu G., Tamada K. & Chen L. B7-H1, a third member of the B7 family, co-stimulates T-cell proliferation and interleukin-10 secretion. Nat Med 5 (**12**), 1365 (1999). 10581077 10.1038/70932

[b7] Butte M. J. *et al.* Programmed death-1 ligand 1 interacts specifically with the B7-1 costimulatory molecule to inhibit T cell responses. Immunity 27 (**1**), 111 (2007). 17629517 10.1016/j.immuni.2007.05.016 PMC2707944

[b8] Park J. J. *et al.* B7-H1/CD80 interaction is required for the induction and maintenance of peripheral T-cell tolerance. Blood 116 (**8**), 1291 (2010). 20472828 10.1182/blood-2010-01-265975 PMC2938239

[b9] Yang J. *et al.* The novel costimulatory programmed death ligand 1/B7.1 pathway is functional in inhibiting alloimmune responses *in vivo*. J Immunol 187 (**3**), 1113 (2011). 21697455 10.4049/jimmunol.1100056 PMC3140607

[b10] Topalian S. L., Drake C. G. & Pardoll D. M. Targeting the PD-1/B7-H1(PD-L1) pathway to activate anti-tumor immunity. Curr Opin Immunol 24 (**2**), 207 (2012). 22236695 10.1016/j.coi.2011.12.009 PMC3319479

[b11] Brahmer J. R. *et al.* Phase I study of single-agent anti-programmed death-1 (MDX-1106) in refractory solid tumors: safety, clinical activity, pharmacodynamics, and immunologic correlates. J Clin Oncol 28 (**19**), 3167 (2010). 20516446 10.1200/JCO.2009.26.7609 PMC4834717

[b12] Herbst R. S. *et al.* Predictive correlates of response to the anti-PD-L1 antibody MPDL3280A in cancer patients. Nature 515 (**7528**), 563 (2014). 25428504 10.1038/nature14011 PMC4836193

[b13] Brahmer J. R. *et al.* Safety and activity of anti-PD-L1 antibody in patients with advanced cancer. N Engl J Med 366 (**26**), 2455 (2012). 22658128 10.1056/NEJMoa1200694 PMC3563263

[b14] Brahmer J. R., Hammers H. & Lipson E. J. Nivolumab: targeting PD-1 to bolster antitumor immunity. Future Oncol 11 (**9**), 1307 (2015). 25798726 10.2217/fon.15.52

[b15] Ghebeh H. *et al.* The B7-H1 (PD-L1) T lymphocyte-inhibitory molecule is expressed in breast cancer patients with infiltrating ductal carcinoma: correlation with important high-risk prognostic factors. Neoplasia 8 (**3**), 190 (2006). 16611412 10.1593/neo.05733 PMC1578520

[b16] Hamanishi J. *et al.* Programmed cell death 1 ligand 1 and tumor-infiltrating CD8 + T lymphocytes are prognostic factors of human ovarian cancer. Proc Natl Acad Sci USA 104 (**9**), 3360 (2007). 17360651 10.1073/pnas.0611533104 PMC1805580

[b17] Thompson R. H. *et al.* Tumor B7-H1 is associated with poor prognosis in renal cell carcinoma patients with long-term follow-up. Cancer Res 66 (**7**), 3381 (2006). 16585157 10.1158/0008-5472.CAN-05-4303

[b18] Liang M., Yang H. & Fu J. Nimesulide inhibits IFN-gamma-induced programmed death-1-ligand 1 surface expression in breast cancer cells by COX-2 and PGE2 independent mechanisms. Cancer Lett 276 (**1**), 47 (2009). 19046800 10.1016/j.canlet.2008.10.028

[b19] Azuma K. *et al.* Association of PD-L1 overexpression with activating EGFR mutations in surgically resected nonsmall-cell lung cancer. Ann Oncol 25 (**10**), 1935 (2014). 25009014 10.1093/annonc/mdu242

[b20] Taube J. M. *et al.* Colocalization of inflammatory response with B7-h1 expression in human melanocytic lesions supports an adaptive resistance mechanism of immune escape. Sci Transl Med 4 (**127**) 127r (2012). 10.1126/scitranslmed.3003689 PMC3568523 22461641

[b21] Parsa A. T. *et al.* Loss of tumor suppressor PTEN function increases B7-H1 expression and immunoresistance in glioma. Nat Med 13 (**1**), 84 (2007). 17159987 10.1038/nm1517

[b22] Zhang P., Su D. M., Liang M. & Fu J. Chemopreventive agents induce programmed death-1-ligand 1 (PD-L1) surface expression in breast cancer cells and promote PD-L1-mediated T cell apoptosis. Mol Immunol 45 (**5**), 1470 (2008). 17920123 10.1016/j.molimm.2007.08.013

[b23] Ghebeh H. *et al.* Doxorubicin downregulates cell surface B7-H1 expression and upregulates its nuclear expression in breast cancer cells: role of B7-H1 as an anti-apoptotic molecule. Breast Cancer Res 12 (**4**), R48 (2010). 20626886 10.1186/bcr2605 PMC2949635

[b24] Zitvogel L., Apetoh L., Ghiringhelli F. & Kroemer G. Immunological aspects of cancer chemotherapy. Nat Rev Immunol 8 (**1**), 59 (2008). 18097448 10.1038/nri2216

[b25] Correale P. *et al.* Chemo-immunotherapy of metastatic colorectal carcinoma with gemcitabine plus FOLFOX 4 followed by subcutaneous granulocyte macrophage colony-stimulating factor and interleukin-2 induces strong immunologic and antitumor activity in metastatic colon cancer patients. J Clin Oncol 23 (**35**), 8950 (2005). 16061910 10.1200/JCO.2005.12.147

[b26] Galluzzi L., Senovilla L., Zitvogel L. & Kroemer G. The secret ally: immunostimulation by anticancer drugs. Nat Rev Drug Discov 11 (**3**), 215 (2012). 22301798 10.1038/nrd3626

[b27] Patel S. P. & Kurzrock R. PD-L1 Expression as a Predictive Biomarker in Cancer Immunotherapy. Mol Cancer Ther 14 (**4**), 847 (2015). 25695955 10.1158/1535-7163.MCT-14-0983

[b28] Kerr K. M. *et al.* Programmed Death-Ligand 1 Immunohistochemistry in Lung Cancer: In what state is this art? J Thorac Oncol 10 (**7**), 985 (2015). 26134220 10.1097/JTO.0000000000000526

[b29] Gao Q. *et al.* Overexpression of PD-L1 significantly associates with tumor aggressiveness and postoperative recurrence in human hepatocellular carcinoma. Clin Cancer Res 15 (**3**), 971 (2009). 19188168 10.1158/1078-0432.CCR-08-1608

[b30] Pardoll D. M. The blockade of immune checkpoints in cancer immunotherapy. Nat Rev Cancer 12 (**4**), 252 (2012). 22437870 10.1038/nrc3239 PMC4856023

[b31] Chen N. *et al.* Upregulation of PD-L1 by EGFR Activation Mediates the Immune Escape in EGFR-Driven NSCLC: Implication for Optional Immune Targeted Therapy for NSCLC Patients with EGFR Mutation. J Thorac Oncol 10 (**6**), 910 (2015). 25658629 10.1097/JTO.0000000000000500

[b32] Afreen S. & Dermime S. The immunoinhibitory B7-H1 molecule as a potential target in cancer: killing many birds with one stone. Hematol Oncol Stem Cell Ther 7 (**1**), 1 (2014). 24398144 10.1016/j.hemonc.2013.09.005

[b33] Yang C. Y. *et al.* Programmed cell death-ligand 1 expression in surgically resected stage I pulmonary adenocarcinoma and its correlation with driver mutations and clinical outcomes. Eur J Cancer 50 (**7**), 1361 (2014). 24548766 10.1016/j.ejca.2014.01.018

[b34] Konishi J. *et al.* B7-H1 expression on non-small cell lung cancer cells and its relationship with tumor-infiltrating lymphocytes and their PD-1 expression. Clin Cancer Res 10 (**15**), 5094 (2004). 15297412 10.1158/1078-0432.CCR-04-0428

[b35] Mu C. Y. *et al.* High expression of PD-L1 in lung cancer may contribute to poor prognosis and tumor cells immune escape through suppressing tumor infiltrating dendritic cells maturation. Med Oncol 28 (**3**), 682 (2011). 20373055 10.1007/s12032-010-9515-2

[b36] Cooper W. A. *et al.* PD-L1 expression is a favorable prognostic factor in early stage non-small cell carcinoma. Lung Cancer-J Iaslc 89 (**2**), 181 (2015). 10.1016/j.lungcan.2015.05.007 26024796

[b37] Antonia S. J. *et al.* Nivolumab (anti-PD-1; BMS-936558, ONO-4538) in combination with platinum- based doublet chemotherapy (Pt-DC) in advanced non-small cell lung cancer (NSCLC). ASCO Meeting Abstracts 32, 8113 (2014).

[b38] Tang Y. *et al.* The association between PD-L1 and EGFR status and the prognostic value of PD-L1 in advanced non-small cell lung cancer patients treated with EGFR-TKIs. Oncotarget 6 (**16**), 14209 (2015). 25895031 10.18632/oncotarget.3694 PMC4546461

