# Erratum: Expression of programmed death ligand-1 on tumor cells varies pre and post chemotherapy in non-small cell lung cancer

**DOI:** 10.1038/srep23850

**Published:** 2016-04-13

**Authors:** Jin Sheng, Wenfeng Fang, Juan Yu, Nan Chen, Jianhua Zhan, Yuxiang Ma, Yunpeng Yang, Yan Huang, Hongyun Zhao, Li Zhang

Scientific Reports 6: Article number: 2009010.1038/srep20090; published online: 01292016; updated: 04132016

The original version of this Article contained an error in the spelling of the author Yan Huang, which was incorrectly given as Yanhuang. This has now been corrected in the PDF and HTML versions of the Article.

