# Supplementary material for: Expression of programmed death ligand-1 on tumor cells varies pre and post chemotherapy in non-small cell lung cancer
Source: Sci Rep. 2016 Jan 29;6:20090. doi: 10.1038/srep20090 (PMC4731819; doi:10.1038/srep20090)

Expression of programmed death ligand-1 on tumor cells varies pre and post chemotherapy in non-small cell lung cancer

Jin Sheng[1,2,3,*], Wenfeng Fang[1,2,3,*], Juan Yu[3], Yunpeng Yang[1,2,3], Yuxiang Ma[1,2,3], Nan Chen[4], Jianhua Zhan[1,2], Zhihuang Hu[1,2,3], Tao Qin[1,2,3], Ting Zhou[1,2,3], Yan Huang[1,2,3], Hongyun Zhao[1,2,3] and Li Zhang[1,2,3]

[1]State Key laboratory of Oncology in South China, Sun Yat-sen University Cancer Center, Guangzhou, P. R. China

[2]Collaborative Innovation Center for Cancer Medicine, Sun Yat-sen University Cancer Center, Guangzhou, Guangdong, China

[3]Department of Medical Oncology, Sun Yat-sen University Cancer Center, Guangzhou, P. R. China

[4]Department of Medical Oncology, the Fifth Affiliated Hospital of Sun Yat-sen University, Zhuhai, Guangdong, China

*These author contributed equally to this work.

Correspondence and requests for materials should be addressed to Li Zhang (zhangli@sysucc.org.cn.)

Supplementary Table S1. Patient characteristics and levels of programmed death-ligand 1 expression on TCs pre and post-NACT.

**Characteristics**	**N (%)**	**H-score of PD-L1 on TCs**			
		**Pre-treatment**	***p***	**After NACT**	***p***
**Age（years）**					
< 56	16 (50.0)	100 (60-120)	0.636	70 (30-87.5)	0.636
≥56	16 (50.0)	90 (70-127.5)		80 (27.5-100)	
**Gender**					
Female	17 (53.1)	90 (65-135)	0.676	60 (25-90)	0.324
Male	15 (47.9)	100 (70-110)		80 (30-110)	
**Smoking**					
Never smoker	22 (68.8)	100 (70-130)	0.337	75 (45-102.5)	0.500
Current or ex-smoker	10 (31.2)	85 (50-112.5)		75 (0-92.5)	
**Pathology**					
Adenocarcinoma	26 (81.3)	100 (70-130)	0.330	80 (45-100)	0.323
Squamous carcinoma	5 (15.6)	70 (50-100)		30 (10-85)	
Others	1(3.1)	NA		NA	
**Stage**					
IIIA (N21)	21 (65.6)	90 (70-110)	0.175	70 (25-85)	0.282
IIIB (T4N0 or 1)	11 (34.4)	120 (60-150)		90 (30-170)	
**Exon**					
Mutated	11 (34.4)	80 (70-140)	0.942	30 (0-100)	0.260
Wild-type	15 (46.9)	100 (60-120)		80 (50-100)	
Untested	6 (18.8)	85 (65-117.5)		65 (77.5-105)	
**NAC regimen**					
Paclitaxel-based	9 (28.1)	90 (50-110)	0.399	70 (25-85)	0.048
Pemetrexed-based	15 (46.9)	100 (70-130)		90 (50-120)	
TKI-based	8 (25.0)	90 (70-120)		40 (0-80)	
**Down-staging**					
Yes	16 (50.0)	100 (67.5-112.5)	0.909	75 (45-102.5)	0.703
No	16 (50.0)	75 (57.5-122.5)		75 (15-100)	
**NACT efficacy**					
Partial response	18 (56.2)	100 (72.5-130)	0.112	75 (22.5-100)	0.925
Stable or progression	14 (43.8)	75 (52.5-117.5)		75 (50-97.5)	
Abbreviations: PD-L1 = programmed death-ligand 1; NACT = neo-adjuvant chemotherapy; TCs = tumor cells; TKI = tyrosine kinase inhibitors.					

Supplementary Table S2. PD-L1 expression on TCs and ICs at baseline and post-NACT. PD-L1 was identified by IHC. The difference of PD-L1 status (IHC score 0, 1, 2 and 3) pre and post-NACT was significant (*p*=0.007) on TCs, but not applied for ICs (*P*=0.337).

	**TCs**			**ICs**		
	**Baseline**	**After NACT**	***p*-value**	**Baseline**	**After NACT**	***p*-value**
**H-score***	95 (70-120)	75 (30-100)	0.005	10 (0-47.5)	0 (0-37.5)	0.474
**PD-L1 positivity**	24 (75%)	12 (37.5%)	0.003	14 (44.8%)	18 (56.2%)	0.319
**IHC score 0**	8 (25.0%)	20 (62.5%)	0.007	18 (56.2%)	14 (43.8%)	0.337
**IHC score 1**	10 (31.2%)	5 (15.6%)		6 (18.8%)	7 (21.9%)	
**IHC score 2**	7 (21.9%)	3 (9.4%)		6 (18.8%)	9 (28.1%)	
**IHC score 3**	7 (21.9%)	4 (12.5)		2 (6.2%)	2 (6.2%)	
Abbreviations: PD-L1 = programmed death-ligand 1; NACT = neo-adjuvant chemotherapy; TCs = tumor cells; ICs = tumour-infiltrating immune cells; IHC = immunohistochemical method. * H-score was presented as median value with Q1-Q3.						

Supplementary Table S3. Changes of PD-L1 expression of TCs pre and post-neoadjuvant NACT and patient characteristics. Abbreviations: *PD-L1*, programmed death-ligand 1; *NACT*, neo-adjuvant chemotherapy; *TCs*, tumor cells; *TKI*, tyrosine kinase inhibitors.

**Characteristics**	**N (%)**	**H-score of PD-L1 on TCs**		
		**Pre-NACT**	**Post-NACT**	***p***
**Age（years）**				
< 56	16 (50.0)	100 (60-120)	70 (30-87.5)	0.053
≥56	16 (50.0)	90 (70-127.5)	80 (27.5-100)	0.034
**Gender**				
Female	17 (53.1)	90 (65-135)	60 (25-90)	0.003
Male	15 (47.9)	100 (70-110)	80 (30-110)	0.395
**Smoking status**				
Never smoker	22 (68.8)	100 (70-130)	75 (45-102.5)	0.027
Current or ex-smoker	10 (31.2)	85 (50-112.5)	75 (0-92.5)	0.065
**Histologic diagnosis**				
Adenocarcinoma	26 (81.3)	100 (70-130)	80 (45-100)	0.018
Squamous carcinoma	5 (15.6)	70 (50-100)	30 (10-85)	0.078
Others	1(3.1)	NA	NA	NA
**Stage**				
IIIA (N21)	21 (65.6)	90 (70-110)	70 (25-85)	0.008
IIIB (T4N0 or 1)	11 (34.4)	120 (60-150)	90 (30-170)	0.196
**Types of EGFR mutation**				
Mutated	11 (34.4)	80 (70-140)	30 (0-100)	0.007
Wild-type	15 (46.9)	100 (60-120)	80 (50-100)	0.083
Untested	6 (18.8)	85 (65-117.5)	65 (77.5-105)	0.916
**NAC regimen**				
Paclitaxel-based	9 (28.1)	90 (50-110)	70 (25-85)	0.050
Pemetrexed-based	15 (46.9)	100 (70-130)	90 (50-120)	0.442
TKI-based	8 (25.0)	90 (70-120)	40 (0-80)	0.018
**Down-staging**				
Yes	16 (50.0)	100 (67.5-112.5)	75 (45-102.5)	0.016
No	16 (50.0)	75 (57.5-122.5)	75 (15-100)	0.110
**NACT efficacy**				
Partial response	18 (56.2)	100 (72.5-130)	75 (22.5-100)	0.004
Stable or progression	14 (43.8)	75 (52.5-117.5)	75 (50-97.5)	0.196

Supplementary Table S4. Disease-free survival analyses based on clinical-pathological features and PD-L1 status around NACT. Abbreviations: *PD-L1*, programmed death-ligand 1; *NACT*, neo-adjuvant chemotherapy; *TCs*, tumor cells; *TKI*, tyrosine kinase inhibitors.

**Characteristics**	**Median DFS (months)**	**Univariate analyses**	***p***	**Multi-variate analyses**	***p***
**Age（years）**					
< 56	25.9	Referent		Referent	
≥56	20.7	1.051	0.92	1.24	0.74
**Gender**					
Female	14.2	Referent		Referent	
Male	25.9	0.87	0.76	0.24	0.13
**Smoking status**					
Current or ex-smoker	14.2	Referent		Referent	
Never smoker	26.0	0.69	0.45	0.14	0.04
**Histologic diagnosis**					
Non-squamous	17.1	Referent		Referent	
Squamous carcinoma	20.7	0.50	0.35	0.74	0.72
**T Stage**					
1	26.0	Referent		Referent	
≥2	12.0	1.70	0.24	3.15	0.09
**N Stage**					
N0	23.1	Referent		Referent	
N1 or 2	12.0	2.63	0.036	12.9	0.002
**Types of EGFR mutation**					
Mutated	25.9	Referent		Referent	
Wild-type	13.7	1.07	0.45	2.10	0.11
Untested	9.6	1.13			
**NACT regimen**					
Paclitaxel-based	25.9	Referent		Referent	
Pemetrexed-based	12.7	1.39	0.71	1.26	0.58
TKI-based	23.1	0.94			
**Change of PD-L1 status**					
Other situation	23.1	Referent		Referent	
Negative to positive	9.6	4.96	0.02	3.88	0.17

Supplementary Figure S1.Kaplan-Meyer curves of PD-L1 negative-to-positive switch and then all other cases. Median disease-free survival was 9.6 months versus 23.1 months, p=0.009.

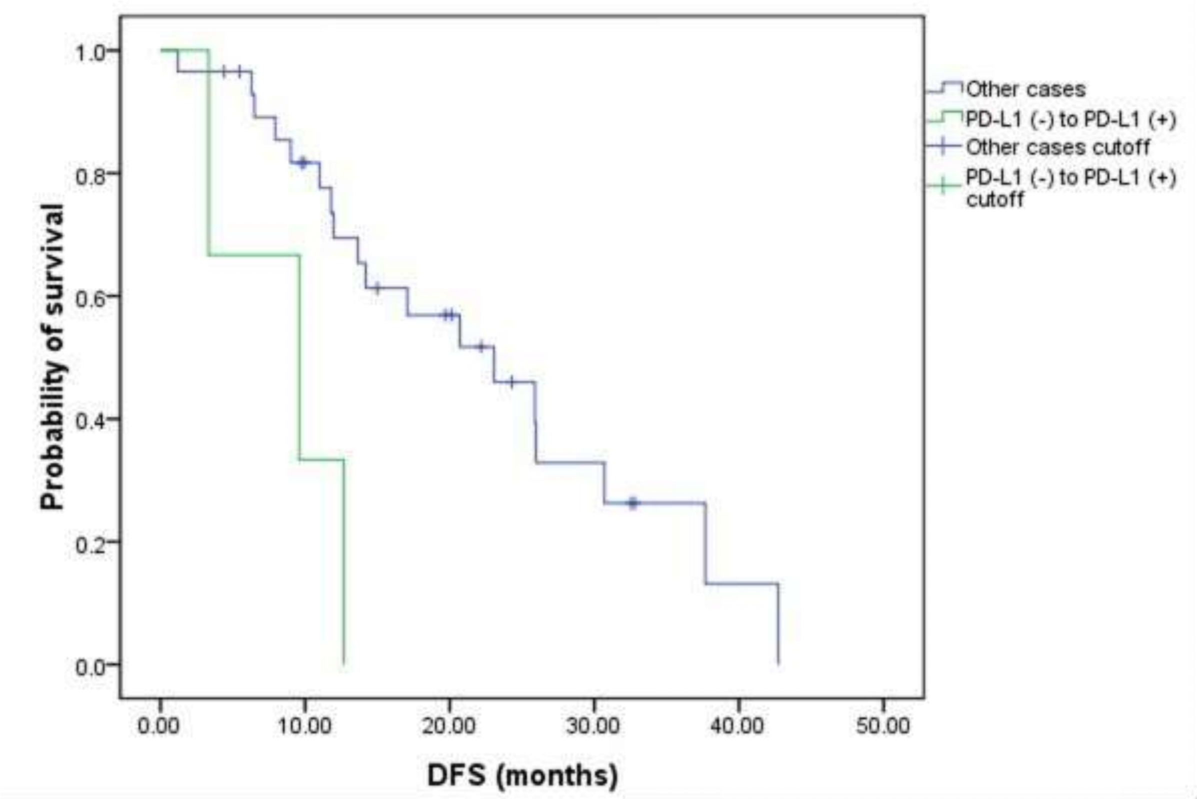

Supplement: Supplementary Files [file srep20090-s1.pdf]
